# Respiratory distress syndrome is the poster child for neonatology

**DOI:** 10.1038/s41390-024-03723-1

**Published:** 2025-01-16

**Authors:** Alan H. Jobe

**Affiliations:** 1 Cincinnati Children’s Hospital Medical Center, Cincinnati, OH USA; 2 University of Cincinnati College of Medicine, Cincinnati, OH USA

## Introduction

**Fig. 1:**
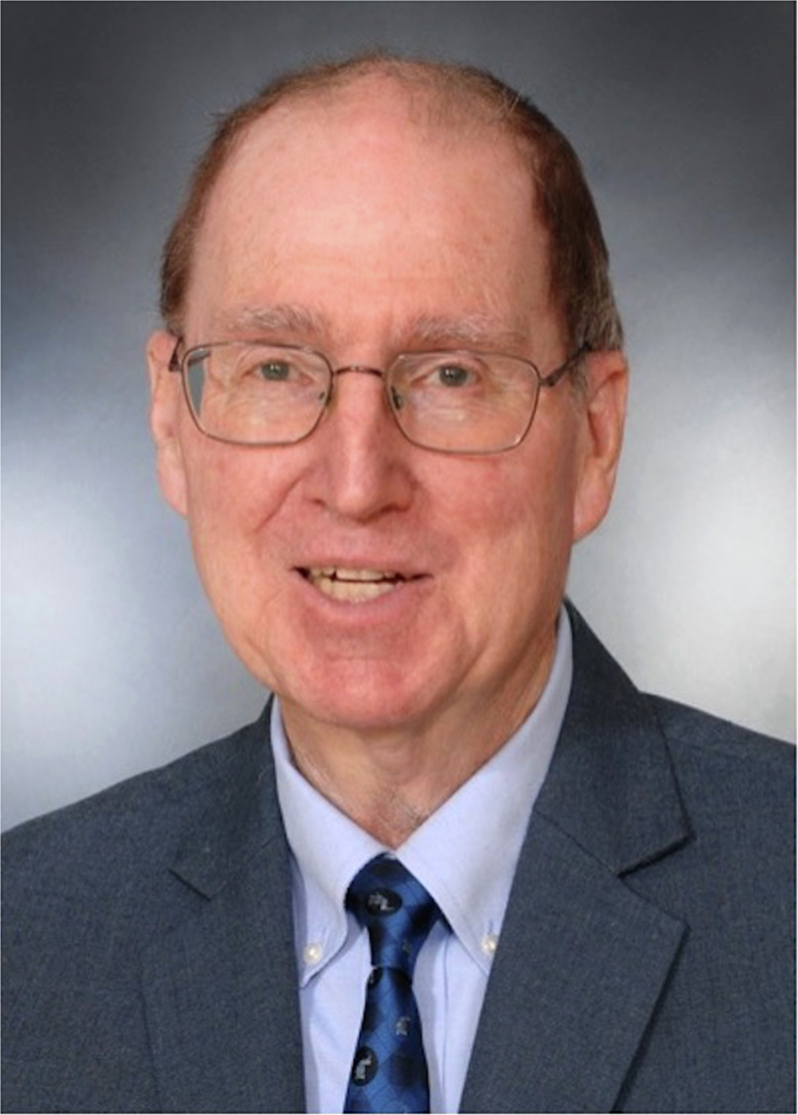
Alan Jobe 4 years into his glioblastoma - November 2023. Picture just prior to the Howland Award (2024).

My goal is to discuss the significant improvement in mortality rates for respiratory distress syndrome (RDS) due to new therapies. In the 1950s and 1960s, mortality rates were approximately 50% (Fig. 1); however, with the introduction of CPAP, surfactant therapy, antenatal steroids, and mechanical ventilation, today’s mortality rate has dropped to less than 2%. This is a remarkable advancement, and the 2% figure may be overestimated due to misdiagnosis of RDS in some instances. My primary interests have been to understand surfactant metabolism and physiology.^1^ I collaborated on this research for over two decades with Machiko Ikegami,^2^ Harris Jacobs,^3^ Andrea Petennazzo,^4^ and Jim Lewis.^5^

I failed in two important life transitions. I flunked the 5th grade, and I got kicked out of hospice care last year since I was doing too well with my glioblastoma (now 4.5 years into diagnosis). The fact that I made it to Toronto to receive the Howland Medal – is surprising. I need to thank my parents for recognizing that I could not read in the 5th grade. They decided to enroll me in a preparatory school, where I repeated the 5th grade and found support to overcome my reading challenges.

I wish to express gratitude to my first scientific mentor, Del Fisher, during my tenure as a faculty member for two decades from 1987 to 1997 at Harbor UCLA. While I was a junior faculty member, I learned a crucial lesson from Del Fisher: that comprehending human diseases necessitates dependable animal models, as conducting mechanistic studies in humans is frequently too difficult. Thankfully, we had access to exceptional animal models for RDS, such as preterm sheep, rabbits, and monkeys. Harbor UCLA provided me with invaluable research space, dedicated time, exemption from extensive clinical duties, and financial support, laying the foundation for my academic journey. Thanks also to the two exceptional junior neonatal faculty members at Harbor UCLA, Jim Padbury and J. Usha Raj, who had their own highly successful academic careers at UCLA. Del’s remarkable achievements, including receiving the Howland Medal in 2001 for his groundbreaking work in fetal endocrinology, were truly inspiring.

## Respiratory distress syndrome (RDS)

**Fig. 2:**
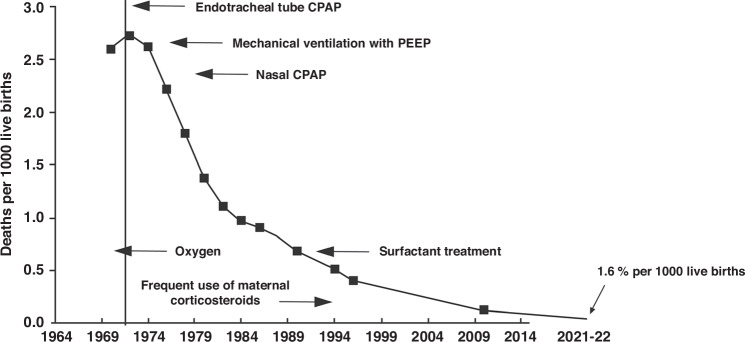
The relationship between mortality and death from respiratory distress syndrome in all births in the United States from 1965 through nine 2023. The death rate was about 50% in the late ‘60 s, and it fell to a low of less than 2% by the last measurements that were made. This estimate is probably a high estimate because very preterm infants are often coded as dying of RDS even though they’re dying of severe prematurity.

**Fig. 3:**
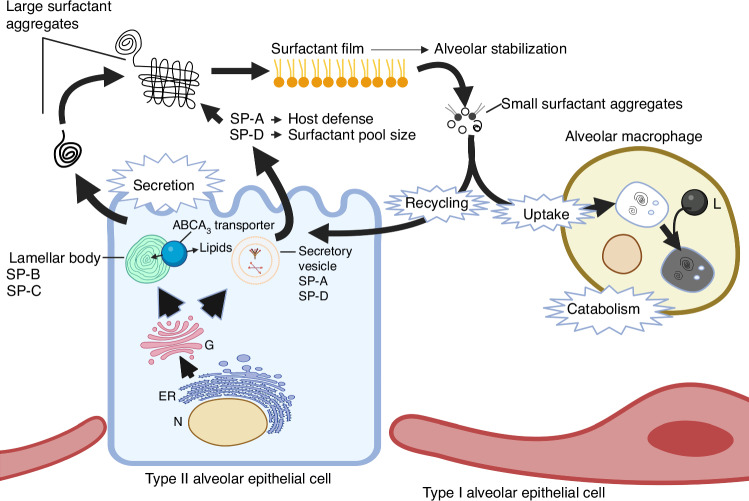
Survey of metabolic pathways of surfactant.^59^

**Fig. 4:**
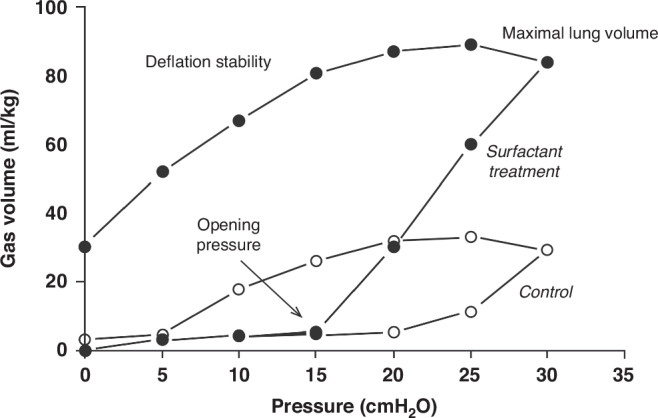
P.V. curves of premature rabbit lungs treated with 100 mg sheep surfactant This figure encapsulates all of the physiological effects of surfactant on the preterm lung. The lower curve shows preterm rabbits treated with nothing as the controls, and the upper curve shows preterm rabbits treated with sheep surfactant. The fall of the opening pressure from about 30 centimeters of water to 15 centimeters indicates that the lung opens at a much lower pressure and to a much higher volume of almost 85 ML per kilogram. It deflates progressively as the pressure drops to retain about 25 ML per kilogram of volume.^11^ (Re-drawn from original data).

**Fig. 5:**
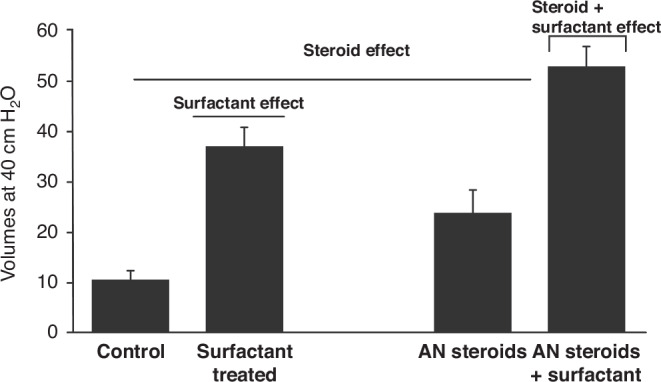
Demonstrates the additive effects of steroids plus surfactant. If you apply 40 centimeters of water pressure to a preterm sheep lung, the lung volume expands only to 10 ml per kilogram. Surfactant treatment opens to about 35 ml per kilogram with antenatal steroid treatment; the lung opens to about 20 mg per kilogram. With both therapies, lung volumes increase to 50 ml per kilogram. (Re-drawn from original data).

Surfactant deficiency is the primary cause of RDS.^6^ To understand RDS more thoroughly, we conducted extensive metabolic research spanning many years to comprehend the mass action dynamics of surfactant components (Figs. 2, 3). This understanding proved pivotal in obtaining FDA approval for surfactant treatment of RDS in 1990. Additionally, our investigations revealed surfactant inactivation as a significant concept in the field (Fig. 4, 5).^7^

RDS is the poster child success story for neonatology. Death from RDS was about 50% in the 1970s when oxygen was the only therapy to treat premature infants with RDS. The latest data for all live births in the U.S. is a mortality <2%; that is a high estimate as many of those deaths are probably miss-coded (Fig. 2).

Based on my experiences working on the Lac – operon at the Salk Institute^8^ and in discussions with Mikko Hallman, a research fellow from Finland,^9^ working with Louis Gluck, MD at the University of California San Diego (UCSD), we realized that there was a lack of information regarding surfactant pool sizes, secretory rates, and the function of surfactants in both adult and preterm lungs (Fig. 3). This gap hindered our ability to assess whether surfactant therapy could be a viable therapeutic option. To better understand Mel Avery’s seminal observation, that preterm infants were surfactant deficient, and the pressure-volume curves were abnormal we studied lung function in preterm animal models (Fig. 4).^10,11^

## Continuous Positive Airway Pressure (CPAP)

Continuous Positive Airway Pressure (CPAP), described by George Gregory,^12^ was also a major area of interest that we studied in preterm lambs.^13^ Treatment of RDS with positive airway pressure was a major contribution to neonatal care, perhaps ever more important for decreasing mortality than surfactant because it simply treated the physiologic abnormality, reversing atelectasis with striking improved oxygenation. We found that surfactant lipid pool size of 4 mg/kg was sufficient to maintain PAO2 in preterm ventilated lambs.^13^ The use of CPAP to change lung physiology was rapidly translated to therapy. Moreover, in collaboration primarily with Noah Hillman, we conducted studies on lung injury using preterm rabbit^14^ and lamb models^15^ to deepen our understanding of the role of CPAP. CPAP for infants was based on Harrison’s^16,17^ physiologic observations in 1986, showing that intubating infants took away their grunt with resultant lowering of their PAO2 values. Extubation resulted in increased oxygenation related to grunting.^16^ They were delivering their own CPAP. Beena Kamath-Rayne and I estimated that in 2011, 70% of the improved outcomes in RDS mortality were related to CPAP alone.^18^

I conducted research on animals alongside Jane Pillow,^19^ Machiko Ikegami,^20^ Noah Hillman,^14^ Graeme Polglase,^21^ Boris Kramer,^22^ and Suhas Kallapur,^23^ demonstrating the efficacy of CPAP.^19^ Clinical data from South Africa indicates that most infants with RDS can be effectively managed solely with CPAP.^24^ Building on this, I believe CPAP is the primary intervention for reducing RDS mortality.

Data that we generated on surfactant metabolism were important for the FDA’s approval of surfactants for treating infants. Lacking was information on surfactant pool sizes, secretion mechanisms, and distribution pathways (Fig. 3). What was unique about surfactants as a drug was their mode of treatment by intratracheal instillation. The surfactants initially assessed were derived from cow or pig lungs. Their effects were not typical of traditional drugs but instead relied on biophysical effects related to surface tension.^25^

We conducted our research using rabbit and sheep models, where we isolated the lipids and proteins from surfactants in rabbits and sheep.^26,27^ With the assistance of Jeff Whitsett at Cincinnati Children’s Hospital, we radio-labeled these components.^28^ Radio labeling was the only method available to trace them at that time. Subsequently, we discovered that the surfactant pool sizes in infants with RDS were small, ranging from 2 to 10 milligrams per kilogram. The surfactant treatments we used were large, up to 100 to 200 milligrams per kilogram, likely needed to address distribution challenges.

The metabolism of surfactant components is interesting. In the newborn and particularly in preterm newborn babies, surfactant components are recycled for re-secretion with an efficiency approaching 90%.^3^ The increase in surfactant pools from synthesis takes about three days before newly secreted surfactant is available in the airspace. Surfactant lipid is lost from the airspace with a half-life of about three days and is similar in animal models and babies, the latter we studied with stable isotopes in Holland.^29,30^ We demonstrated the impact of surfactant replacement on static pressure-volume curves using preterm rabbits.^31^ Fig. 3 shows all of the major effects of surfactant, which are to lower the opening pressure, increase the maximal lung volume massively and maintain lung stability on deflation.^32^

## Surfactant inactivation

Over several years, we conducted significant research on surfactant inactivation (Fig. 5), demonstrating that most inflammatory mediators and serum proteins can inhibit surfactant function. Our experiment on surfactant inhibition involved collecting airway samples from infants who required intubation for treatment of RDS (Fig. 5). We obtained tracheal-bronchial suction samples from these infants as they underwent mechanical ventilation. These airway samples exhibited high minimum surface tensions, exceeding 20 dynes/cm, while samples from mature infants showed very low surface tension values (Fig. 5).^7^ Intriguingly, when we layered these airway samples over a sucrose gradient, we were able to isolate surfactant with excellent surface activity. Additionally, we found that the supernatants from these samples could inactivate sheep surfactant. This discovery, I believe, was the first demonstration of surfactant inactivation as a significant issue in RDS. Our findings were published in 1983 by our group at Harbor UCLA (Fig. 5).^7^

## Antenatal steroids

**Fig. 6:**
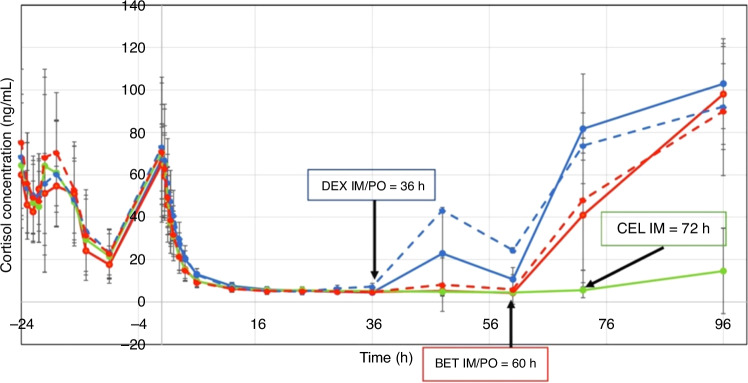
These are values from 48 nonpregnant women from Bangalore, India. They were administered 1/4 of the dose of betamethasone-phosphate or maternal dexamethasone-phosphate to assess the pharmacokinetics in reproductive-age nonpregnant women. Betamethasone-phosphate or betamethasone acetate plus betamethasone phosphate (Celestone), were each given orally or I.M. The curve in green represents the present drug used for antenatal steroid therapy. To assess suppression in adrenal function, we observed a rapid loss of plasma cortisol that persisted for more than a week. The women who received 6 mg betamethasone acetate received one dose; therefore, women in labor who routinely received two 12 mg doses are likely to have suppression of adrenal function for at least two weeks.^34^

Subsequently, we explored the synergistic interactions between antenatal steroids and surfactants (Fig. 5).^33^ Our findings indicated that the dosage of steroids administered to women may be excessively high, potentially providing a risk for neurodevelopmental impairments in infants (Fig. 6).^34^ The World Health Organization (WHO) and the Bill and Melinda Gates Foundation are currently conducting trials to test a low-dose strategy in numerous low-resource locations based on our animal studies.^35^

We have studied antenatal steroids in a series of experiments spanning 40 years carried out in Perth, Australia, with John Newnham, the head of the Obstetrics department, and young investigators who collaborated with me to set up these experiments.^36–39^ I would devise the protocols and travel to Australia every summer to conduct them. Tim Moss, Jane Pillow, Peter Sly, Graham Polglase, and Matt Kemp were among those who worked with me on these projects. Currently, we are continuing some of these experiments with Matt Kemp.^38^

**Fig. 7:**
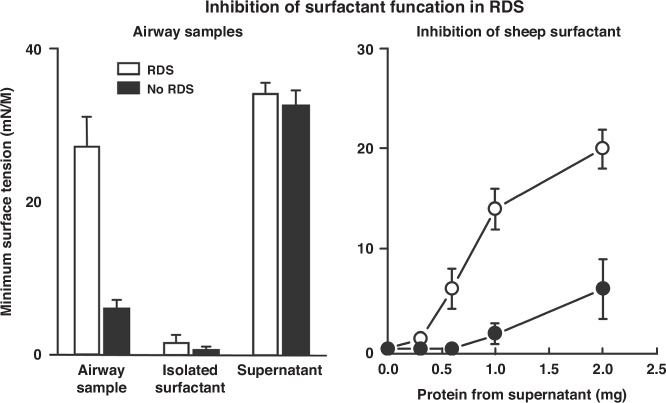
The effect of airway samples from babies intubated for RDS ^7^It’s a very old study showing that the surface tension of material isolated from RDS babies was very high, above 20 dynes per centimeter. If you layer the airway samples over a sucrose gradient, you can recover surfactant with good surface properties. If one adds supernatants of lavage material to surfactant, its activity is inhibited.^7^ This figure shows that babies with RDS have active surfactant material, but its activity is inactivated.

The improved outcomes in babies today are largely due to the use of CPAP administration of antenatal steroids and surfactant replacement therapy (Fig. 7). The majority of preterm infants now at risk from RDS receive antenatal steroids. I was privileged to conduct a pharmacokinetic study in Bangalore, India, funded by the Bill and Melinda Gates Foundation.^34^ One of the major problems with any of the current standard-of-care treatments of antenatal steroids is that dose-response curves or pharmacokinetic studies were not performed. These studies should have been done 50 years ago before the wide use of this therapy.^32^

We have done extensive studies in sheep and monkeys, and we find that the present therapy for antenatal steroids (Celestone), is a mixture of antenatal betamethasone phosphate and betamethasone acetate. The acetate component causes prolonged release (Fig. 6). To our surprise, we discovered that in 48 nonpregnant women of reproductive age in Bangalore, the steroid levels remained pharmacologically effective even after seven days.^34^ The initial normal biological curve for endogenous cortisol is suppressed when given only 1/4 of the dose of steroids used clinically for women at risk for preterm labor. Cortisol levels are strongly suppressed and do not start recovering endogenous cortisol levels within at least a week. At present, women at risk for preterm birth are given two doses, indicating that they are likely suppressed for 14 days. Thus, women in preterm labor are likely in adrenal suppression. So, our goal for the future is that there are to study these pathways regulating lung maturation in preterm infants. One is antenatal steroids, and the other is chorioamnionitis. In models using either endotoxin or IL-1 one beta, both cause lung maturation. Surprisingly, infection is much more potent and effective than antenatal steroids. Virtually all fetal sheep respond to steroids, as do fetal monkeys.^32^ We are working with a team of cell and molecular biologists at Cincinnati Children’s Hospital Medical Center to test if there is a common pathway that links these two mechanisms that induce lung maturation and may lead to new therapies that do not require steroids. Understanding the cell and molecular signaling that induces maturation without glucocorticoids and their possible toxic effects may be developed to prevent RDS.^37^

I want to recognize Mont Liggins, who passed away in 2010, as the individual who initially proposed the use of antenatal steroids to enhance fetal lung maturation to reduce the occurrence of RDS.^40^ His groundbreaking work began with a publication in 1972 involving approximately 250 patients, demonstrating decreased mortality and RDS. It is of historical interest that subsequent reanalysis of this work revealed no reduction in mortality^41^ but a significant decrease in RDS. Perhaps, we may be using incomplete data when discussing the utility of antenatal steroid therapy for RDS.

I would also like to highlight Suhas Kallapur, who has conducted extensive research regarding fetal inflammation using samples from monkeys and sheep in Australia.^42^ He has contributed significantly to the understanding of how lung development and injury occur in newborns, particularly in the context of infection and inflammation. He is the Division Chief at the University of California, Los Angeles (UCLA). Boris Kramer from Maastricht, Netherlands, has also collaborated with me for over 20 years.

## Conclusion

I want to conclude with two quotes; one comes from William Silverman, one of my heroes in neonatology.^43^ The first one is from Archie Cochrane’s quote.

“My colleagues, in their devotion to their patients, evoke my admiration but also remind me of the demands of families that nothing be left undone for their preterm infants. In the future, I hope clinicians will abandon the margin of impossible and settle for reasonable probability.“^44^

The final quote is from William Silverman:

“Callahan recognizes that the vulnerability to sickness and death can only be reduced, never be vanquished,” and he paraphrased that in a typical Bill Silverman way, “Life is a universally fatal sexually transmitted disease.“^45^

## Additional considerations regarding neonatology

Three existential risks to neonatology, if our goals are to improve normal outcomes at the limits of survival of extremely preterm infants, are to be accomplished.^46^

1. Developmental Origins of Health and Disease (DoHAD). Life Before Birth: The challenges of fetal development.^47^
2. Unanticipated late risks of antenatal steroids.^35^

A new neonatal disease, “Dysmaturation Syndrome,” is related to the survival of very preterm infants.

- Smaller brains.^48–50^
  i. Abnormal neurodevelopmental outcomes.^51^
  ii. Increased infection in the first year of life.^52^
  - Early aging.^50^
  - Early onset of Chronic Obstructive Pulmonary Disease COPD.^53–55^
  - Early onset of heart failure.^56,57^
  - Early onset kidney failure.^58^
  - Early onset aging could be called a Dysmaturation Syndrome.^50^
